# A comprehensive review of the roles of T-cell immunity in preeclampsia

**DOI:** 10.3389/fimmu.2025.1476123

**Published:** 2025-02-06

**Authors:** Xu Peng, Ibeh Chinwe Oluchi-Amaka, Joanne Kwak-Kim, Xiuhua Yang

**Affiliations:** ^1^ Department of Obstetrics, The First Hospital of China Medical University, Shenyang, China; ^2^ Reproductive Medicine and Immunology, Obstetrics and Gynecology, Clinical Sciences Department, Chicago Medical School, Rosalind Franklin University of Medicine and Science, North Chicago, IL, United States; ^3^ Clinical Immunology Laboratory, Foundational Sciences and Humanities, Microbiology and Immunology, Chicago Medical School, Rosalind Franklin University of Medicine and Science, North Chicago, IL, United States

**Keywords:** pregnancy, preeclampsia, T cells, Treg cells, Th17 cells

## Abstract

Preeclampsia (PE) is an obstetrical disorder that occurs after the 20th week of gestation. It is recognized as one of the “Great Obstetrical Syndromes” and principally contributes to maternal morbidity and mortality. PE has been associated with a range of immune disorders, including a preponderance of T helper (Th) 1 over Th2 cells and imbalanced levels of Th17 and T regulatory cells (Tregs). During pregnancy, T cells safeguard the placenta against immune rejection and aid embryo implantation while involved in pregnancy complications, such as PE. Promoting alloantigen-specific Treg cells is a potential preventive and therapeutic strategy for PE. However, ensuring the safety of mothers and infants is of the utmost importance since the risk-benefit ratio of reproductive and obstetric conditions differs significantly from that of immune diseases that pose a life-threatening risk. In this review, we systematically summarize the roles of T-cell immunity in the peripheral blood, reproductive tissues, and at the maternal-fetal interface of PE patients. Furthermore, the recent therapeutic approaches centered on targeting T cell immunity in PE are critically appraised.

## 1 Introduction

Preeclampsia (PE) is a disorder that occurs during pregnancy, with a global incidence rate of 2-8%. Recognized as one of the “Great Obstetrical Syndromes,” it principally contributes to maternal mortality (1). Clinical manifestations, including hypertension (BP ≥ 140/90 mmHg) and proteinuria, manifest after the 20th week of gestation to define this condition (2). Furthermore, PE may be correlated with additional maternal and obstetrical complications, such as impairment of placentation, intrauterine growth restriction, preterm labor, aberrant liver function, acute renal failure, and hematological abnormalities (3, 4). Currently, preventive measures are limited to lifestyle modifications and the use of aspirin, whereas management consists solely of childbirth. Although early pregnancy induction is often required to safeguard the health of the mother in cases of PE, premature birth can have significant adverse effects on neonatal health. For example, respiratory morbidity in neonates has been observed in preterm infants, which exhibited a 4.4-fold increase in comparison to full-term infants (5).

Currently, knowledge holds that PE develops in two stages: an impaired trophoblast invasion and remodeling of spiral arteries, along with changes in the immune system in the early maternal-fetal environment. During the latter phase of pregnancy, systemic inflammation occurs in the maternal body (6). Overall, significant pathogenic factors include the presence of inflammatory processes (7–9), the lack of maternal tolerance towards the fetus (10–12), and cardiovascular maladaptation in the mother (13). Noticeably, PE is associated with a range of immune disorders, including higher activity of neutrophils, monocytes, and natural killer (NK) cells, dysregulated cytokine secretion, a preponderance of T helper (Th) 1 cells relative to Th2 cells, imbalanced levels of Th17 and T regulatory cells (Tregs), and the manifestation of autoimmunity (14–18). Tregs, which have decreased levels of CD127 and increased levels of CD4, CD25, cytotoxic T lymphocyte antigen-4 (CTLA-4), CD45RA, HLA-DR, and forkhead transcription factor 3 (FoxP3), are particularly important in preventing the development of detrimental immune responses and promoting tolerance throughout pregnancy (19).

Over the last two decades, considerable investigation has indicated that T cells exert notable influence on both healthy and unhealthy pregnancies, albeit with the exact characteristics of these influences remaining obscure. Disputing the notion that decidual T cells universally endanger fetal survival due to the “allograft” placenta, distinct T cell subsets contribute to determining pregnancy success or failure. Treg cells safeguard the placenta against immune rejection and aid in embryo implantation. In contrast, others, such as Th1 or Th17 cells, have been reported to be involved in pregnancy complications, such as PE.

In this review, we systematically summarize the roles of T-cell immunity in the peripheral blood, reproductive tissues, and at the maternal-fetal interface of patients diagnosed with PE and further substantiate the notion that T cell regulation may effectively mitigate the detrimental prognosis of PE for both the mother and neonate, ultimately leading to improved pregnancy outcomes.

## 2 T cells in normal pregnancy

In the human decidua of the first trimester, T cells comprise a range of 10-20% of the overall leukocyte count, comprised of 30-45% CD4^+^ and 45-75% CD8^+^ (20). Afterward, the proportion of decidual T cells increases, eventually encompassing 40-80% of all leukocytes at term (21). The regulation of normal reproductive functions depends on CD4^+^ T cells and the immune factors they generate. Suitable T cell reactions regulate the fertilization and embryo development, as well as the initial development of the placenta and angiogenesis (22–26). Based mainly on the production of cytokines and surface markers, CD4^+^ T helper cells are categorized as Th1, Th2, Treg, Th17, and recently Th22 cells (27). A controlled transition to Th1 responses occurs during the peri-implantation phase; these responses are involved in immune surveillance and prevent an overabundance of trophoblast invasion (28). Transitioning towards Th2 cells following placental implantation is imperative to ensure and advance the development of a healthy embryo and placenta by promoting allograft tolerance. It entails the reduction of Th1 and Th17 cells through the secretion of interleukin (IL)-13, IL-4, and IL-17 (28). The Th1/Th2 paradigm during normal pregnancy is that Th1 immune response dominates in the first trimester. However, in the second and third trimesters, the maternal immune system shifts towards Th2 immune response (28–30).

CD8^+^ T cells are the prevailing immune cells in the decidua during gestation, essential for fostering fetal-maternal tolerance. Effector memory CD8^+^ T cells (CD8^+^ EM cells) comprise the majority of decidual CD8^+^ T cells (dCD8^+^ T cells); these cells are thought to possess the capability to induce fetal rejection. Naive CD8^+^ T cells (CD8^+^ N cells), on the other hand, constitute the main parts of peripheral CD8^+^ T cells (pCD8^+^ T cells) (31). In comparison to pCD8^+^ EM cells, dCD8^+^ EM cells express greater quantities of interferon-γ (IFN-γ) and IL-4, whereas perforin and granzyme B are less abundantly expressed (31, 32). Programmed cell death-1 (PD-1) was discovered to be highly expressed on Tregs, CD8^+^ T cells, and NKT-like cells (33–35). A notable up-regulation of programmed death ligand-1 (PD-L1) was observed in immune cells situated at the maternal-fetal interface, as well as extravillous trophoblasts (EVT) and syncytiotrophoblasts (ST) (36–39). Fetal resorption is increased in mouse models when the PD-1/PD-L1 signal is inhibited; this suggests that this pathway is important for maintaining immune tolerance in the decidua (40). Accordingly, immune tolerance to fetal antigens is sustained in pregnancy by regulating the response of decidual CD8^+^ T cells, although these cells maintain the capability to eliminate virus-infected cells (41).

**Figure 1:**
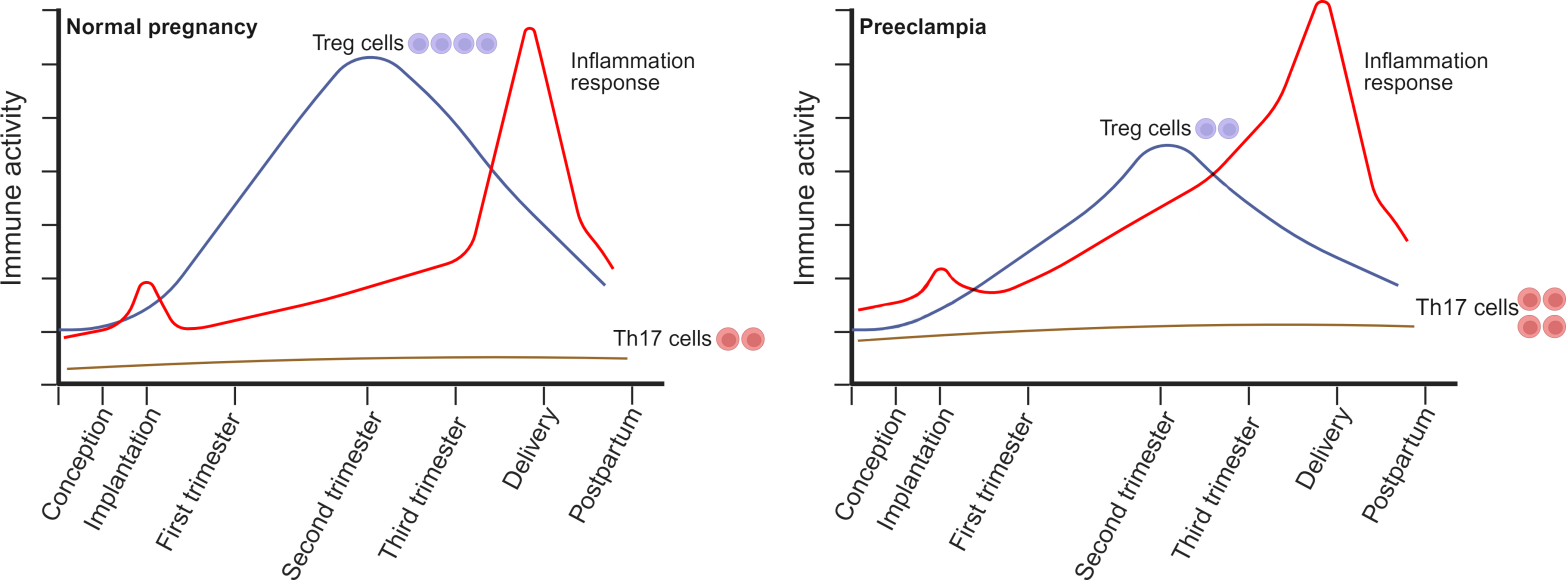
The alterations in the circulating composition of Treg/Th17 cells during normal pregnancy and preeclampsia (PE) occur throughout the peri-conception, various stages of pregnancy, and at delivery. During a healthy pregnancy, Treg cell levels rise during early pregnancy, peak in mid-pregnancy, and then decline progressively after delivery, returning to slightly elevated levels compared to pre-pregnancy levels. In contrast to the non-pregnant state, the quantity of Th17 cells remains comparatively low during the entirety of the pregnancy and does not endure substantial changes. PE is correlated with reduced quantities, compromised suppressive capabilities, or instability of Treg cells. This may be attributed to the limited ability of Treg cells to facilitate normal placentation and regulate the elevated inflammation frequently observed in PE. Furthermore, the quantity of Th17 cells in expectant women with PE could be considerably greater than that of healthy controls. Generated using BioRender.com.

CD4^+^CD25^+^ Treg cells are essential in protecting the fetus from rejection due to their potent ability to suppress immune responses (42). Many Treg subtypes resembling induced type 1 regulatory (Tr1) cells have been detected in the initial and terminal human decidua (43). These subtypes have exhibited the capability to impede the proliferative ability of effector T cells and increase the secretion of IL-10 (44). Healthy pregnancy was related to a rise in CD4^+^CD25^+^ Tregs in both rodents (45) and humans (46). There is a rise in the circulating levels of CD4^+^CD25^+^ cells in the first trimester, reaching the highest value in mid-pregnancy and then declining after childbirth to levels slightly higher than those before pregnancy (46) (**Figure 1**). A few weeks prior to delivery, a discernible reduction in CD4^+^CD25^high^ Treg cells is observed (47). Treg cells are locally enriched in the decidua (48, 49). Researchers observed similar proportions of Tregs in the decidua basalis throughout normal pregnancies, while noting a rise in the decidua parietalis from mid pregnancy to late pregnancy (49).

Tregs serve multiple functions, including inhibiting immune system activity, mitigating inflammation, and remodeling of blood vessels to promote successful embryo implantation in the decidua (10, 50). During pregnancy, peripheral Treg cells and uterine Treg cells are generated in reaction to fetal antigens. Peripheral Treg cells inhibit the production of the pro-inflammatory cytokines (IFN-γ, tumor necrosis factor (TNF)-α, IL-2, and IL-12), creating a microenvironment with anti-inflammatory properties (51–53). Uterine Treg cells suppress the expansion of Th1 and Th17 cells, thereby averting their assault on the semi-allogeneic fetus. Inhibiting the immune activity of effector T cells to fetal alloantigens, uterine Tregs have diagnostic features of inhibitory T cells, including elevated expressions of CTLA-4, IL-10, CD25, and transforming growth factor (TGF)-β (54–57). Moreover, Tregs are essential in preventing invariant NKT (iNKT)-induced miscarriage (56). In support of a successful pregnancy, Tregs also aid in the maintenance of a suppressive immune phenotype in other cell types, including macrophages, dendritic cells (DCs), and uterine NK (uNK) cells (50). Conversely, interaction between CD14^+^ myelomonocytic cells and decidual NK cells initiates a sequence of events that facilitate the production of Treg cells and inhibit the immune response (58).

In contrast, Th17 cells constitute an additional subgroup of CD4^+^ T cells that contribute to facilitating inflammatory responses (59). Th17 cell-produced IL-22 and IL-17 contribute to the eradication of pathogens and the induction of inflammation in autoimmune disorders, respectively (60, 61). The regulation of Th17 cell development and differentiation is subject to the influence of the retinoic acid-related orphan receptor γt (RORγt), as well as a multitude of positive and negative factors (62, 63). In a healthy pregnancy, CD4^+^ T cells comprise the majority of IL-17-producing cells in the circulation and decidua (64, 65). Comparatively, the percentage of IL-17^+^ lymphocytes in the decidua is evidently greater than that in the periphery of first-trimester pregnant women (64). The circulating quantity of Th17 cells exhibited no variation during the entire gestational period (66). Research has documented that pregnant women exhibit reduced quantities of CD4^+^ IL-17^+^ T cells and an increased proportion of FoxP3^+^ Tregs to IL-17^+^ CD4^+^ T cells during a healthy pregnancy compared to non-pregnant females (14). IL-17 could augment the invasiveness of JEG-3 cells, which are choriocarcinoma cell lines, and substantially increase progesterone secretion *in vitro* co-culture models (67, 68). Another study found that IL-17 levels remained in a similar range throughout the whole pregnancy, but the average was higher in the third trimester (69). This suggests that elevated IL-17 levels may contribute to the initiation of labor and/or inflammation. Further research involving humans and *in vivo* experiments is required to ascertain whether the existence of Th17 cells is a cause or an effect of effective pregnancy establishment.

To summarize, the accumulation of Tregs is critical for maintaining a healthy semi-allogeneic pregnancy during its early stages. The proportion of Tregs increases during the initial and subsequent trimesters of gestation, and through a multitude of mechanisms, Tregs promote the well-being of the fetus. While significantly more Th17 cells are present in the decidua in early pregnancy compared to the second and third trimesters, these cells secrete IL-17, facilitating the invasion and proliferation of trophoblasts.

## 3 Effector T cells in PE

Women with PE have an increased systemic effector T-cell pool, as demonstrated by either a higher number/proportion or a greater degree of activation (70–73). Patients diagnosed with PE exhibited elevated circulating concentrations of CD4^+^ T cells compared with those undergoing a healthy pregnancy (74). The elevation in CD4^+^ T cells could potentially be ascribed to the proliferation of memory T cells since the percentage of this subgroup was greater in women with PE relative to those who conceived successfully; contrarily, the percentage of naive T cells was diminished (72). Aberrant function of CD8^+^ T cells has also been observed in cases of PE compared to healthy pregnancies (73). A rise in the cytotoxicity of CD8^+^ T cells was found in preeclamptic women relative to non-preeclamptic women; this may have been due to a reduction in Treg-mediated inhibition (70). In addition to the heightened cytotoxic activity, patients with PE exhibited a significant increase in the proportion of peripheral microparticles originating from cytotoxic T cells compared to controls who were not pregnant (75).

As stated previously, the majority of studies have indicated that women with PE have increased T-cell activity. However, there have been several reports indicating contradictory findings. Compared to women with a normal pregnancy, patients with PE had decreased peripheral T-cell counts (76, 77). According to these reports, while the numbers of CD8^+^ and CD4^+^ T cells were comparable between normal and preeclamptic pregnancies, PE patients exhibited significantly lower percentages of CD4^+^ memory, CD4^+^ EM, and CD4^+^ central memory (CM) subgroups than normal pregnancies (78). Reduced T cell populations may indicate a decline in specific subgroups instead of a decline in the T-cell population as a whole. Undoubtedly, pregnancies impacted by PE exhibited a dramatic decrease in the percentage of circulating CD4^+^ HLA-G^+^ T cells (79) and diminished levels of soluble HLA-G in maternal plasma (80). This observation holds significance since HLA-G, which is predominantly detected in neonatal tissues (81) and functions to enhance immune tolerance (82, 83), can be detected in a distinct subgroup of T cells that possess immunosuppressive characteristics (84, 85). Hence, specific peripheral T cell subsets may be reduced in the context of PE, which contrasts with the elevated abundance of inflammatory T cells documented in other reports.

Efforts to examine the functionality of effector T cells at the uteroplacental interface have produced contradictory results, potentially due to variations in patient cohorts, experimental approaches, and other specific characteristics. Flow cytometric (86) and immunohistochemical (87) analyses of decidual samples revealed a reduction in the percentage of T cells in PE cases compared to women who experienced preterm delivery or full-term childbirth, respectively. Conversely, an immunohistochemistry method identified a prominent abundance of CD8^+^ T cells in the decidua of individuals diagnosed with PE compared with those without (88). CD8^+^ T cells and total T cells were more abundant in placental bed biopsies of women with PE than normal controls (89). Additionally, a greater percentage of CD8^+^ T cells was identified in placental tissues from pregnancies impacted by PE (90). The modifications in local effector T cells associated with PE may be influenced by distinct aspects: the flow cytometry analyses of early-onset and late-onset PE revealed a decrease in the percentages of CD4^+^ central-memory (CM) T cells and CD8^+^ regulatory-(Foxp3^+^) memory cell (CD45RO^+^) T cells compared with normal pregnant women, and early-onset PE showed higher proportions of activated CD4^+^ and CD8^+^ cells compared to late-onset PE in the decidua parietalis (91). Hence, it is evident that the changes in the local T-cell linked to PE are not solely determined by specific subsets, as early-onset PE is frequently accompanied by more significant immunological changes that may be more severe. The elevated prevalence of decidual effector T cells from women with PE could potentially be attributed to additional regional factors that trigger a secondary immune response.

Potentially, acute atherosis associated with PE or other placental histological abnormalities may enhance the infiltration of T cells. More T cells were detected in the decidual samples of preeclamptic females with acute atherosis than those without this pathological change (92). The significance of considering possible confounding variables, such as placental lesions, should be emphasized when assessing immune alterations in women with PE.

The reports mentioned above collectively indicate that PE is distinguished by activated T cells in the decidua or placenta and maternal circulation. The prevalence of systemic inflammation and a decrease in the number and functionality of Tregs and immunosuppressive HLA-G^+^ T cells are probable factors that impact T cell activation. Notably, women who develop PE at an early stage of pregnancy appear to have increased T-cell activities; however, other situations that can induce T-cell-mediated pathogenesis, such as acute atherosis, should also be evaluated.

## 4 The Th1/Th2 paradigm in PE

The hypothesis regarding immune regulation during pregnancy, suggesting the conversion of the maternal immune response from Th1 to Th2, has been widely accepted for a long time (71, 93, 94). This model is built on the observation that the reaction triggering an antigen presented to a Th0 lymphocyte in a non-pregnant woman will be influenced, at least partially, by the cytokine environment surrounding this lymphocyte. For example, a cytokine microenvironment containing higher expressions of IL-12, IL-18, and IFN-γ will support the generation of Th1 cells, which release TNF-α, IL-2 and IFN-γ, and facilitate the stimulation of other cells such as cytotoxic T cells. On the contrary, a milieu characterized by elevated concentrations of IL-10 and IL-4 will induce the secretion of Th2 lymphocytes. Additionally, the response of Th2 is inhibited by the activity of Th1 cytokines.

PE is distinguished by an imbalance of the Th1/Th2 immune systems, with Th1-type immunity more prevalent in the peripheral circulation (15, 71, 95–101) (**Table 1**). Additionally, cytotoxic T-lymphocytes (CTLs) can be classified as type 1 or 2 subgroups (102, 103). Type 1 cells are distinguished by the inclusion of the IL-18 receptor on the cell surface, whereas type 2 cells exhibit a cell membrane protein resembling IL-1R (104). It has been proposed that the shift towards a Th1 dominance during pregnancy is responsible for the compromised placental function seen in PE. An inquiry observed an increase in the synthesis of IFN-γ, IL-2, and TNF-α in peripheral blood mononuclear cells (PBMCs) from patients with PE. Furthermore, a noteworthy correlation was revealed between the mean blood pressure and Th1 cytokines (105). In another study, 20 PE patients and 20 normotensive counterparts were recruited (106). They discovered that women with PE had a Th1 polarization shift and a Th2 reduction in their peripheral blood profiles. This was linked with elevated concentrations of TNF-α and IFN-γ and lower expressions of TGF-β1 and IL-10, compared with healthy pregnant women (106). These alarmins may cause disease development by modulating CD4^+^ T cells and encouraging the secretion of pro-inflammatory cytokines, thereby inducing innate and adaptive immune responses (106). An additional study examined the concentrations of Th1/Th2 cytokines in peripheral blood lymphocytes and CD3^+^ T, CD4^+^ Th, and CD8^+^ Tc cells in women with PE (107). When comparing PE to a normal pregnancy, an increased count of CD4^+^ lymphocytes was observed (107). While the Th1/Th2 shift was not detected in PE involving CD3^+^ cells, it might be evident in CD4^+^ and CD3^-^ lymphocytes (107).

**Table 1:** The Th1/Th2 cytokine imbalance in preeclampsia (PE).

Th1/Th2 cytokines	Peripheral blood	Maternal-fetal interface
TNF-α ↑	(15, 16, 105, 250–255)	(256, 257)
TNF-α →		(110, 111)
IFN-γ ↑	(15, 98, 101, 105, 106, 258)	(108, 256, 259, 260)
IFN-γ →	(99, 261)	
IL-1 ↑		(256)
IL-2 ↑	(95, 97, 98, 105)	
IL-2 →		(110, 111)
IL-6 ↑	(252–254, 262–264)	(256, 257)
IL-6 ↓	(15)	(108, 259, 265)
IL-6 →		(110)
IL-8 ↑	(124, 252–254, 261, 264)	(257)
IL-12 ↑	(99, 100, 266)	
IL-12 →	(261)	
IL-12 ↓		(108, 259)
IL-18 ↑	(267)	(267)
TGF-β ↑	(268)	
TGF-β ↓	(76)	
TGF-β1 ↑	(262)	
TGF-β1 ↓	(106, 115, 255)	(256)
IL-4 ↓	(15)	
IL-4 →	(95, 115, 261)	
IL-5 ↓	(15, 269)	
IL-10 ↓	(15, 95, 98, 106, 115, 252, 255, 258, 269, 270)	(108, 110, 257, 259)
IL-10 ↑	(253, 262)	
IL-10 →		(111)
Th1:Th2 ratio ↑	(71, 96, 124, 253, 268)	(111)

↑, increase; ↓, decrease; →, no significant change.

In patients with PE, reinforcement of Th1 reactions is evident not only in the periphery but also at the maternal-fetal interface (108). *In vitro* trophoblast cultures derived from term placentas of preeclamptic patients exhibited a profound reduction in IL-10 expression compared with cultures from normal pregnancies (109, 110). In PE, the placental ratios of TNF-α/IL-10 and IL-2/IL-10 were substantially elevated than in a healthy pregnancy (111). In PE, PD-1 expression was decreased in CD8^+^ T cells (112, 113), suggesting further activated CD8^+^ T cells. Compared to normal pregnant women, PE patients exhibited elevated levels of T-bet, Th1 transcription factor and decreased expressions of GATA-3, Th2 transcription factor in circulating and decidual T cells (114, 115).

## 5 Treg/Th17 paradigm in PE

### 5.1 Treg/Th17 imbalance in the peripheral blood of PE patients

Numerous studies have investigated the relationship between PE and peripheral blood Treg populations. Some research investigations utilized flow cytometry to examine the populations of Tregs (77, 106, 115–137), whereas others employed qPCR (62, 114, 138, 139). Pregnant women with PE have decreased numbers of circulating Tregs (14, 77, 117, 120, 123–125, 127, 132, 134, 140–144), including CD4^+^CD25^+^ FoxP3^+^ T lymphocytes (125), and CD8^+^CD25^+^FoxP3^+^ cells (138). However, other studies revealed that there was no significant difference in the peripheral proportion of Treg cells between the normal group and the PE group (136, 145, 146). Treg cells might have a diminished suppressive capacity in PE patients (77, 118, 120, 140, 142).

In patients with PE, the proportions of Treg subtypes are distinct from that of healthy expectant women. In comparison to normal pregnant females, the frequency of fully functional effector Tregs (CD4^+^ FoxP3^+^ CD45RA^−^) was lower in patients with PE. Conversely, naive Tregs (CD4^+^ FoxP3^+^ CD45RA^+^) were unchanged (116). In one study, a distinct subset of Treg cells that express HLA-G was characterized (147). These cells, which are hypo-proliferative and thymic-derived, do not show expression of FoxP3 and CD25 molecules. These entities are identified in instances of multiple sclerosis, HIV-1 infection, and transplantation (148). Compared to normal pregnant women, the proportions of peripheral CD4^+^HLA-G^+^, CD8^+^HLA-G^+^, and CD4^+^CD25^+^CD127^low^ cells are diminished in PE patients (77). Likewise, an additional investigation demonstrated an evident reduction in the circulating CD4^+^HLA-G^+^ T cells in individuals with PE compared to healthy pregnant women (79). Moreover, healthy pregnant women have a significantly higher percentage of circulating CD4^+^CD127^low^CD25^+^, CD4^+^FoxP3^+^, and CD4^+^CD25^high^ cells compared to patients with PE (14). Furthermore, an increased Th17/Treg ratio was identified in PE by researchers via an examination of the proportions of CD4^+^CD25^+^CD127^+^ and CD4^+^ IL-17^+^ cells (122). In addition, the quantity of CD45RA^+^CD31^+^ recent thymic emigrant Tregs are diminished in patients with PE (119).

In PE cases, the inhibitory function of Treg cells diminishes in tandem with their diminished quantity (**Table 2**). It has been discovered that CD4^+^CD25^+^CD127^low/neg^ Treg cells isolated from PE women via magnetic sorting showed a diminished suppressive activity in comparison to Treg cells isolated from healthy expectant individuals (140). In addition, CD4^+^CD25^+^ Treg cells obtained from PE patients via magnetic activated cell sorting (MACS) failed to impede the growth of CD4^+^CD25^−^ responder cells (120). Nevertheless, contradictory studies have been reported that the inhibitory capacity of the Treg cell subset was comparable between PE patients and healthy pregnant controls (14, 126).

**Table 2:** The Treg/Th17 immune imbalance in preeclampsia (PE).

Immune pathway	Peripheral blood	Maternal-fetal interface
Treg	Decreased Treg percentage (141, 143)	Decreased Treg proportion (141)
	Depressed suppressive ability (120, 140)	Reduced Treg clonal expansion (173)
	Decreased FoxP3 mRNA (114, 122)	Lower FoxP3 mRNA at delivery (114)
		Lower IL-10 protein expression (109)
Th17	Increased Th17 cells (120)	
	Increased RORγt mRNA (122)	
	Increased IL-17 level (123, 156)	

Several studies, however, failed to identify any distinctions between the populations of Treg cells in preeclamptic patients and those of healthy pregnancies. However, these studies used frozen PBMCs with a small sample size (145) and investigated CD4^+^CD25^+^ T lymphocytes without specific Treg markers (146). Minimal variation was observed in the quantity and ratio of Treg cells between women with normal pregnancy and PE (146). Additionally, an independent investigation revealed no distinction in the population of activated and resting Treg cells among women with severe and early-onset PE, pregnant women who did not have PE, or non-pregnant females (149). However, an analysis of functional and migratory Treg markers (CCR4 and CTLA-4) revealed that untreated preeclamptic individuals had higher percentages of CTLA-4^+^ and CCR4^+^ resting and inactivated Treg populations than healthy pregnant controls. It is worth noting that ten of eighteen PE patients in this study (149) were treated with glucocorticoids, which may affect the experimental results. It has been reported that glucocorticoids could affect the number and phenotype of Treg cells (150–152). Therefore, it is necessary to select PE patients who have not received glucocorticoids for further research.

The divergent results can likely be attributed primarily to the various definitions of Treg cells. A notable concern pertains to the expression of the critical phenotypic markers, namely CD25 and FoxP3, upon stimulation of conventional CD4^+^ T cells. Determining the precise quantity of “authentic” Treg cells in conditions marked by systemic T-cell stimulation, such as PE, is thus complicated. Diverse flow cytometric methods and markers have been devised to prevent the inclusion of non-suppressive activated Treg cells, which could potentially generate erroneous outcomes. These encompass the identification of suppressive Treg cells characterized by reduced CD4 expression (CD4^dim^) relative to conventional CD4 cells (153), as well as diminished or nonexistent expression of the IL-7 alpha receptor subunit CD127 (154). Additionally, the use of CD45RA helps distinguish between suppressive resting Treg cells (FoxP3^dim^CD45RA^+^) and non-suppressive, activated FoxP3 expressing T helper cells (FoxP3^dim^CD45RA^-^) (155).

The dysregulation linked to PE has been illustrated by modifications in transcription factors of T-cells. RORγt and FoxP3 are transcription factors that contribute to the development of Th17 and Treg cells, respectively (122). Ribeiro et al. (115) revealed that early-onset PE women had a greater proportion of CD4^+^ T cells expressing the RORc transcription factor and a consequential decline in the percentage of Treg cells expressing FoxP3, indicating more severe early-onset PE. Additionally, the mRNA expressions of FoxP3 and RORγt in peripheral T cells of patients with PE were higher and lower, respectively, in comparison to healthy expectant women (114, 122).

Altered concentrations of circulating cytokines may influence the equilibrium between Th17 and Treg cells, thus potentially contributing to the pathogenesis of PE. Pro-inflammatory responses result from the stimulation of chemokines (CXCL1, CXCL2, CCL20), cytokines (IL-6 and TNF-α), and inflammatory factors (the complement system and acute-phase proteins) by IL-17 (156). Consequently, IL-17 could facilitate the proliferation of Th17 cells. Circulating IL-17 concentrations are significantly elevated in PE women relative to normal pregnant females and non-pregnant controls (156). Furthermore, IL-17 concentrations in the plasma of pregnant women with severe PE are higher than those of healthy pregnant women (123). Another group, nevertheless, failed to identify a statistically significant difference in serum IL-17 concentrations between pregnant women with PE and those without complications (157). Furthermore, considering the capacity of IL-6 and IL-1β to stimulate the transformation of Treg cells into Th17 cells (158, 159), it is conceivable that heightened concentrations of these cytokines in PE could trigger the transformation from Treg to Th17 cells (160). It has been found that PE induces a rise in IL-6 and IL-1β production (161). IL-6 expression was found to be elevated in chorionic villus sampling (CVS) tissues of women who subsequently develop PE accompanied by fetal growth restriction (FGR) (162). Increased levels of IL-6 expression promote Th17 generation and undermine the stability of Tregs, while concurrently reducing the quantity of alternatively activated M2 macrophages and T cell markers (163).

Variations in the expression of apoptotic molecules contribute to the premature deletion of Tregs, which may account in part for the diminished quantity of Tregs observed in patients with PE (117). Compared with normal pregnancies, the percentage of Tregs expressing the anti-apoptotic molecule Bcl-2 was considerably diminished in PE patients. Furthermore, there was a significant rise in the expression of pro-apoptotic molecule, Bax, in Tregs of PE patients (117). These findings indicate that the presence of PE may increase the vulnerability of Tregs to apoptotic cell death, which is consistent with the reduced Treg counts observed in this specific clinical scenario. Indeed, the signatures of Tregs and effector T cells were found to be differently regulated in women with PE (164). A consistent reduction in STAT5 signaling in Th1 cells was observed in individuals who subsequently experienced PE (164). IL-2/STAT5 signal contributes to the differentiation of T helper (165) and Treg (166) cells, and it also potentially inhibits Th17 differentiation (167). Furthermore, an increase in the p38 signaling pathway, which is essential for Tregs to exert their suppressive function (168), was observed in Tregs from women who were carrying a healthy pregnancy, but not in those who were diagnosed with PE (164). These results suggest the potential utilization of specific signal pathways in maternal blood for the evaluation of PE.

### 5.2 Treg/Th17 imbalance in the deciduae of PE patients

Various studies have revealed decreased Treg cells and increased Th1 and Th17 immune responses in women with PE using various modalities (**Table 3**). There have been numerous hypotheses regarding the decrease in Tregs observed in women with PE. An initial factor to consider is the potential reduction in decidual Treg differentiation (169). The evidence that peripheral DCs from pregnant women with PE have a stronger ability to stimulate Th1/Th17-like T-cell responses than those from normotensive controls supports this view (170). In addition, alterations in circulating DCs were related to lower peripheral Treg levels in patients with PE (134) and elevated Th17 cell levels in women with early-onset PE (171). Furthermore, the insufficient maturation of decidual lymphatic vessels in PE might impede the penetration of immune cells into this region (172). In fact, a correlation between the number of Tregs and the density of lymphatic vessels in the decidua was established, suggesting that in cases of PE, the entry of circulating Tregs into the decidua may be obstructed (172). Lastly, a study investigating the T-cell receptor (TCR) repertoires of Tregs in the decidua discovered that the proportion of clonally-expanded Tregs decreased obviously in PE pregnancies (173), indicating that the inability of decidual effector Treg cell populations to undergo clonal expansion may be associated with the onset of PE.

**Table 3:** Studies about the Treg/Th17 imbalance at the maternal-fetal interface in preeclampsia (PE) patients.

Authors and year of publication	Race	Experimental group	Control group	Samples	Methods	Results	Conclusions
Y Sasaki et al, 2007 (141)	Japanese and Polish	38 PE patients	40 normal controls	Placental beds	Immunohistochemistry	The proportion of FoxP3^+^ cells within CD3^+^ T cells of PE patients was lower compared with those from normal controls.	The reduced number of Treg cells in PE may affect immune tolerance in pregnancy.
Zhou Jianjun et al, 2010 (114)	Chinese	15 PE patients	15 healthy pregnant women	Decidua	Flow cytometry	PE patients had significantly lower mRNA expressions of FoxP3 and higher mRNA expressions of RORc.	Reduced expression level of FoxP3 mRNA may lead to the dominant Th1 immune response in PE.
Liu Xiaoqian et al, 2011 (271)	Chinese	16 PE patients	18 normal pregnant women	Placenta	qRT-PCR	FoxP3 mRNA expressions were lower in PE cases.	The decrease of Treg cells is associated with the pathogenesis of PE.
Kristen H Quinn et al, 2011 (272)	American	14 late-onset severe PE, and 12 early-onset severe PE	14 healthy term pregnant women	Decidua	Immunohistochemistry	The number of Treg cells was lower in early-onset PE cases compared to late-onset preeclamptic cases and normal pregnant women.	Early-onset PE has a unique pathogenesis, including immune abnormalities.
Behrouz Gharesi-Fard et al, 2016 (256)	Iranian	15 PE patients	15 normal pregnant women	Decidual and chorionic layers of the placentas	qRT-PCR	The mRNA expressions of FoxP3 and GATA-3 in the decidua of PE were significantly reduced, while T-bet mRNA expressions were increased. PE chorionic samples showed significantly lower FoxP3 mRNA expressions and elevated mRNA expressions of RORγt.	T cell immune imbalance in placental tissues can affect normal pregnancy.
Tina A Nguyen et al, 2017 (118)	Caucasian, Hispanic, Black, and Asian	16 PE patients	30 healthy pregnant women	Uteroplacental interface	Flow cytometry	There was no statistically significant difference in the percentage of Treg cells between the two groups.	Treg cells in PE patients did not show a decrease in suppressive ability.
Sayaka Tsuda et al, 2018 (173)	Japanese	7 PE patients	12 normal pregnant women in the third trimester	Decidua	Flow cytometry	Clonally expanded populations of effector Treg cells reduced in PE patients compared with healthy controls.	Failure of clonal expansion of populations of decidual effector Treg cells may lead to the occurrence of PE.
Zhang Yonghong et al, 2018 (273)	Chinese	41 PE patients	67 healthy pregnant women	Decidua	qRT-PCR and western blot	The mRNA and protein levels of FoxP3 were lower in PE compared to normal pregnant women, while the RORγt expression levels were higher.	There was a Treg/Th17 imbalance at the maternal-fetal interface in PE.
Martina Orlovic Vlaho et al, 2020 (274)	Bohemia	13 mild PE, 15 severe PE patients	19 healthy term pregnant women	Decidua basalis	Immunohistochemistry and double immunofluorescence	The number of CD25^+^ FoxP3^+^ cells was decreased in both PE groups.	The reduced immune response in PE may affect Treg cells in the decidua.
Sarah Meister et al, 2022 (178)	German	32 PE patients	34 normal controls	Decidua	Immunohistochemistry	There was a significant decrease in FoxP3 positive cells in PE decidua.	PE patients showed reduced numbers of decidual resident Tregs.

Currently, there are some innovative methodologies attempting to explore the molecular mechanisms underlying the onset of PE, including single-cell RNA sequencing (scRNA-seq). In one study, researchers conducted scRNA-seq on the placenta and decidual tissues of individuals with late-onset PE as well as those in a healthy pregnancy (174). It was discovered that the cells in the decidua that do not express extracellular matrix (ECM) are primarily immune cells, which consist of two subtypes of T cells (T1 and T2) (174). T2 cells show the expression of Treg marker genes, and there is a decrease in the down-regulation of the GO term “regulatory T cell differentiation” in T2 cells within the PE group. The pathway for “positive regulation of cytokine production” and several pathways for interleukin production show decreased activity in T cells, suggesting a significant impairment of their regulatory functions in late-onset PE (174). Another study examined transcriptomic changes specific to different cell types through unbiased scRNA-seq of placental samples, including two individuals with PE and two normotensive pregnant women (175). This comprehensive analysis involved 29,008 cells across 11 distinct cell types, encompassing trophoblasts and immune cells (175). They found that compared with normal pregnancies, there are significant differences in the gene expressions of GZMA, CD3E1, and CD3G in T cells from PE placental tissues, suggesting that these genes may be involved in the pathogenesis of PE (175). These findings provide a novel molecular theoretical foundation for the treatment of PE.

Treg cell dysfunction or a deficiency in quantity within the decidua has been linked to diminished invasion of EVT and ineffectual remodeling of spiral arteries. These factors collectively disrupt placental growth and ultimately result in placentation disturbance (176, 177). Therefore, it is not difficult to hypothesize that a deficiency of Treg cells during pre- and peri-conception could act as an initial catalyst for a cascade of events culminating in impaired placental development and shallow placentation, which ultimately manifest as conspicuous symptoms of PE later in pregnancy. Furthermore, in PE, Tregs undergo apoptosis at the feto-maternal interface, and galectin-2 (Gal-2) appears capable of inhibiting this process (178). Moreover, modifications in the activity of uNK cells may result in a potential dysregulation between Th17 and Treg cells. It has been shown that uNK cells communicate with CD14^+^ monocytic cells to induce the development of Treg cells and that IFN-γ produced by decidual NK cells can impede Th17 cells; these effects result in reduced inflammation and increased maternal-fetal tolerance (58, 179).

Treg cells possess a unique combination of anti-inflammatory and immune-regulatory properties that endow them with formidable capabilities to support placental development, facilitate the adaptation of maternal blood vessels, suppress inflammation, and preserve maternal acceptance of the fetus. As a result, enhancing the functionality of Tregs and augmenting their quantity in order to regulate the immune response may represent a viable therapeutic strategy for these pregnancy-related complications, including PE. However, in the context of human reproduction, any experimental evaluation of a method to stimulate Tregs must be conducted extremely cautiously and in strict adherence to robust principles of clinical trial design. Ensuring the safety of mothers and infants is of the utmost importance, and it is critical to recognize that the risk-benefit ratio of reproductive and obstetric conditions differs significantly from that of immune diseases that pose a life-threatening risk. Consideration should be given to the potential adverse effects of artificially increasing maternal Treg cells, such as a diminished capacity to combat pathogens (180) or immune surveillance for cancer (181). Notwithstanding the necessity for a comprehensive assessment of various approaches and identification of appropriate patient cohorts, the advanced immune therapy must take precedence to control over reducing adverse health outcomes and mortality rates associated with PE.

## 6 Memory T cells in PE

Memory T cells are a distinct subgroup of T cells that develop subsequent to a previous encounter with an antigen (182). Cytokines including IL-23, IL-7, and IL-15 influence the survival, function, and cytokine production of memory T-cells (183–185). CD4^+^ and CD8^+^ cell lineages both demonstrate the ability to recognize CM and EM cells (182, 186–188). Research on memory T cells in PE has been scant and inconsistent to date. Two investigations identified elevated circulating numbers of the general CD4^+^ memory T cells in PE cases compared with normal pregnant women (72, 144). However, whether the observed differences pertained to the EM or CM cell subset was not specified. An alternative investigation revealed a significant elevation in the quantities of DR^low+^CD45RA^-^ Tregs and DR^high+^CD45RA^-^ Tregs among pregnant women diagnosed with PE compared to the pregnant women with normal blood pressure (140). Nevertheless, DR^low+^CD45RA^-^ and DR^high+^CD45RA^-^ Tregs were not classified as memory Tregs in this investigation, notwithstanding the widespread usage of CD45RA^-^ as a memory marker. In addition, the percentages of mature naive Tregs and circulating recent thymic immigrant (RTE) were lower in women with PE. Conversely, the proportion of memory Treg cells increased, suggesting a possible disruption in the differentiation of peripheral Treg cells (119, 189). PE patients had lower percentages of circulating CD4^+^ memory, CD4^+^ EM, and CD4^+^ CM subgroups than normal pregnancies (78). Furthermore, comparing healthy pregnant females and PE women, the levels of CD4^+^ EM cells in peripheral blood and lymphocytes isolated from intrauterine biopsies obtained during cesarean section were identical (118). However, caution should be exercised in interpreting these findings due to the inability to ascertain the precise cell origin extracted from the biopsy. Furthermore, clonally expanded CD8^+^ EM cells in the deciduae of PE women exhibit decreased PD-1 expression compared with normal pregnancies (112), indicating that the existence of local expansion of effector T cells may be due to a decrease in their suppressive function in PE patients.

## 7 Th22 cells in PE

Th22 cells are known for their production of IL-22. An *in vitro* experiment has shown that IL-22 could prevent premature birth caused by inflammation (190). RORγt and T-bet have opposing effects on the differentiation of Th22 cells from naive Th cells; RORγt promotes it while T-bet inhibits it. On the other hand, Th22 cells have the potential to differentiate into either Th1 or Th2 cells. The Th22 cells showed significant flexibility in generating IFN-γ under conditions that promote Th1 immunity or in an inflammatory environment rich in IFN-γ in living organisms (191). Th22 cells exhibit the ability to enhance IL-13 production when exposed to a Th2 environment (191). The presence of decidual IL-22^+^ Th17/Th2 and Th17/Th0 subsets in normal pregnancy suggests that these cells are important for embryo implantation (192). In patients with severe PE, there was a notable rise in the circulating levels of Th22 cells and IL-22 compared with healthy pregnant individuals (123).

## 8 T cells in cord blood of PE women

The proportion of Treg cells in cord blood from normal pregnancies has been examined in three studies; the percentages are 2.63-8.94% (193), 4.0-10.0% (194), and 2-3% (195). The normal ranges for T lymphocyte subgroups in cord blood from healthy full-term neonates have been reported: 15.40% to 70.06% for Th cells (CD3^+^/CD4^+^); 9.65% to 34.28% for cytotoxic T cells (CD3^+^/CD8^+^). The reference interval for Treg cells spanned from 0.35% to 9.07%, in contrast to the reference interval for adult peripheral blood (1.64% to 6.45%) (196).

Several investigations have explored the modifications in umbilical cord T cells of women with PE. A shift in the percentages of Th1/Th2 and Th17/Treg in the direction of inflammation with unchanged Th1 and Th17 cells and reduced Th2 and Treg cells was noticed (197). Furthermore, an obvious decrease in the proportion of CD4^+^ T cells was observed in the umbilical cord blood of neonates delivered by mothers with PE (198). Compared to healthy pregnant individuals, this decline was accompanied by a substantial decrease in the proportion of FoxP3^+^ Treg, specifically within the FoxP3^lo^ populations (198). Additionally, the proportions of CD4^+^CD25^high^FoxP3^+^ and CD4^+^FoxP3^+^ Treg were evidently lower, whereas the proportion of CD4^+^CD25^low^ was prominently increased in the cord blood of neonates born from preeclamptic women (199). Hence, it suggests that PE is associated with aberrant fetal immunity, manifesting as a reduction in Treg levels within the cord blood.

## 9 Potential therapies for PE targeting T cell immunity

### 9.1 Inhibiting IL-17 and reducing Th17 cells

**Figure 2:**
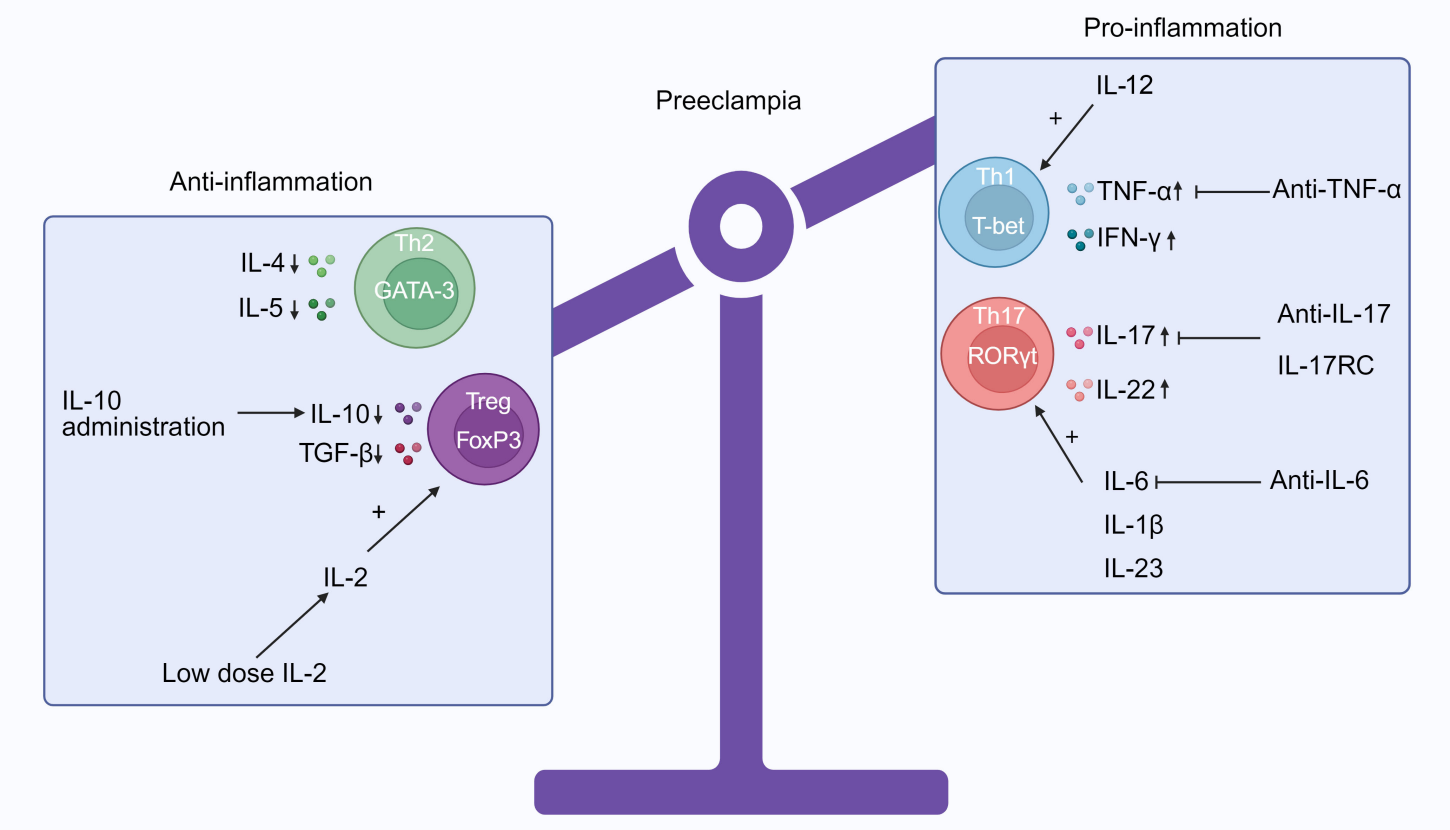
The immune imbalance between Th1/Th2 and Treg/Th17 in preeclampsia (PE) and potential treatments that target CD4^+^ T cells. Patients with PE have a propensity to Th1 and Th17 phenotypes, as indicated by the cytokine profiles in their peripheral blood. These phenotypes are distinguished by elevated concentrations of TNF-α, IFN-γ, IL-17, and IL-22 and reduced expressions of IL-4, IL-5, IL-10, and TGF-β. Additionally, IL-6, IL-1β, and IL-23 can induce the differentiation of Treg cells into Th17 cells. Increased levels of IL-12 will support the generation of Th1 cells. Potentially, regulating the proportion of pro- to anti-inflammatory CD4^+^ T cells could be implemented to prevent PE by promoting healthy placental development and maintaining a viable pregnancy. It is also possible to repress particular cytokines, such as IL-6 via anti-IL-6, IL-17 via IL-17RC and anti-IL-7, and TNF-α via anti-TNF-α. It is known that IL-2 directly regulates Treg homeostasis by enhancing their survival, proliferation, and function. Consequently, low-dose IL-2 can augment the quantity and durability of Treg cells. In addition, IL-10 administration, which increases Treg cell differentiation and Th2 immune response and significantly decreases circulating Th1 and Th17 cells, might be a potential treatment option (Produced with BioRender.com).

IL-17 is important to the body’s response to bacterial infections and inflammation. In addition to compromised tolerance in conditions such as PE, it has been associated with contact dermatitis, autoimmune disorders, and organ rejection following transplantation. Secukinumab, a monoclonal antibody that specifically targets IL-17, has been employed in treating contact dermatitis (NCT02778711) and discoid lupus erythematosus (NCT03866317) to correct the Th17 imbalance. Tibulizumab (LY3090106) is a tetravalent antibody currently under development for the treatment of Sjögren’s disease (NCT04563195). It functions as a dual antagonist against B cell activating factor and IL-17. The long-term use of recombinant mouse IL-17 receptor C (IL-17RC), a soluble receptor that inhibits IL-17, reduced uterine perfusion pressure (RUPP) in rats as well as reduced levels of circulating IL-17 and placental Th17 cells (200). Thus, the prospective efficacy of IL-17RC in the treatment of pro-inflammatory effects mediated by IL-17 in PE was demonstrated (**Figure 2**).

### 9.2 Reducing IL-6

In addition to inhibiting immunosuppressive Tregs and Th2 cells, IL-6 enhances the differentiation of Th17 and cytotoxic T cells, thereby leading to inflammation in PE (201). IL-6 inhibitors, such as anti-IL-6 receptor mAbs (sarilumab and tocilizumab) and anti-IL-6 mAbs (siltuximab), have the potential to drive the development of inexperienced CD4^+^ T cells away from inflammatory Th1 and Th17 subsets (202). Clinically, anti-IL-6 therapy has been used to treat atherosclerosis (203) and rheumatoid arthritis (202). Incorporating strategies to decrease IL-6 for the management of PE may potentially promote the transformation of naive CD4^+^ T cells into Treg and Th2 cells, as opposed to Th1 and Th17 cells.

### 9.3 IL-10 administration

In the RUPP model, IL-10 administration via infusion through an osmotic mini-pump implanted intraperitoneally results in modest reduction in blood pressure as well as a substantial reduction in the levels of circulating Th1 and Th17 cells, an up-regulation of Treg cell differentiation, and the restoration of TNF-α expressions to normal (204). Culturing naive CD4^+^ T cells with IL-10-producing DCs could enhance the differentiation of Treg cells (204), a process that potentially alleviates PE. This study (204) indicate that IL-10 may function as an innovative treatment for PE.

### 9.4 Neutralizing TNF-α

TNF-α, a potential target for modulating CD4^+^ T cells, is produced by Th1 and Th17 cells. TNF-α expressions in PE could be considerably increased, reaching 2-3 times higher than in pregnancies with normal blood pressure (176, 205). These elevated levels have been linked to complications such as gestational hypertension, compromised endothelial function, and unfavorable obstetric outcomes (206). In addition to its essential function in facilitating successful implantation and placental development, TNF-α also functions as an inhibitor of CD4^+^ T cell proliferation via a mechanism dependent on IL-10 (11). By inhibiting effector T cells and repressing the activity of TNF-α, anti-TNF-α neutralizing mAbs promote the development of Tregs (207). The British Association of Dermatologists suggests that TNF-α inhibitors may be used during pregnancy, when large cohort studies that are well-designed, with a follow-up of over 10 years are performed in the future (208). According to the European Crohn’s and Colitis Organization, it is acceptable to utilize TNF-α inhibitors during the third trimester of pregnancy (208). Considering its perceived safety, anti-TNF-α could potentially function as a treatment option for PE by increasing the quantity of Treg cells and restoring the Th1/Th2 and Th17/Treg ratios.

### 9.5 Progestogens

17-hydroxyprogesterone caproate (17-OHPC), a progesterone derivative, is deemed secure for application in obstetrics. In a PE rat model, 17-OHPC induced an elevation in infant weight and a reduction in uterine artery resistance index (UARI) (209). Implementing an intervention such as 17-OHPC to enhance the current treatment for PE patients may have positive effects on both the mother and child. Furthermore, by activating glucocorticoid receptors, progesterone induces the proliferation of Treg cells and advances apoptosis in conventional T cells (210). Consequently, the use of the orally-administered progestogen or dydrogesterone may have the potential to alleviate the adverse pregnancy outcomes in women with PE (211–213).

### 9.6 Intravenous immunoglobulin G

IVIg is employed in the treatment of various immune-regulated disorders, organ transplantation, and systemic inflammatory conditions by virtue of its capacity to inhibit the proliferation of DCs, increase concentrations of anti-inflammatory IL-10, and diminish the functionality of pro-inflammatory T cells (214). Moreover, IVIg improves pregnancy outcomes by stimulating Tregs’ responses (215). IVIg therapy may, therefore, be contemplated as a prospective therapeutic intervention for PE. In women with a history of recurrent pregnancy loss (RPL) and unexplained infertility, the prevalence of PE was comparable with normal pregnant women when they were treated with prednisone and IVIg (216).

### 9.7 Vitamin D

VD has the ability to affect many cell types, such as immune cells, specifically CD4^+^ T cells (217). The circulating concentrations of VD were considerably diminished in pregnant women with PE in comparison to those without (218–220). Furthermore, in comparison to pregnant women with VD levels above 20 ng/mL, those with VD deficiency (25(OH) D < 20 ng/mL) have a five-fold increased risk of developing PE (220). Patients with PE who received VD supplementation exhibited decreased levels of IFN-γ, TNF-α, IL-17, IL-6, and IL-23, whereas IL-10 and TGF-β levels elevated (218, 219). VD exerts its immunomodulatory effects through multifaceted mechanisms, including inhibition of Th1 and Th17 cells, up-regulation of Th2 cell expression, and facilitation of Treg cell proliferation (221–225). Therefore, VD could potentially be suggested as a strategy for regulating the systemic inflammatory response in PE. Moreover, exposure to sunlight (226) and exercise (227) contribute to the maintenance of Treg homeostasis. Nevertheless, the precise mechanisms of VD in human Treg cells remain obscure.

### 9.8 Aspirin

Receiving low-dose aspirin from 11-14 weeks reduced the occurrence of preterm PE in high-risk women compared to those who received a placebo (228). This discovery indicates that giving aspirin early on may decrease the likelihood of PE, potentially by enhancing placental development. A study done on mice revealed that aspirin greatly increased the percentage of functional CD4^+^CD25^+^FoxP3^+^ Treg cells (229). Patients with RPL associated with antiphospholipid syndrome (APS) showed elevated levels of serum cytokines, T cell phenotypes, and transcription factor gene expressions indicative of Th1 responses, while those indicative of Th2 responses were decreased (230). The imbalance between Th1 and Th2 could be restored in patients who responded well to the combination of low-molecular weight heparin (LMWH) and aspirin treatment (230). Moreover, aspirin functions through the production of aspirin-triggered lipoxin, which inhibits the effects of antiphospholipid antibodies on the migration of human trophoblasts and their interactions with endothelial cells (231). In other diseases, aspirin can exert therapeutic effects by regulating T cell function. One example is the potential for aspirin to mitigate the progression of atherosclerosis through restoring balance to the Th17/Treg axis (232). Additionally, aspirin could potentially suppress the autoimmune reaction in atherosclerosis by promoting the expansion of CD4^+^CD25^+^FOXP3^+^ Treg cells (233). In studies on experimental autoimmune encephalomyelitis (EAE), aspirin could enhance Tregs while reducing Th1 and Th17 responses (234, 235). This includes an increase in the levels of FoxP3 and IL-4 in T cells, as well as inhibiting the differentiation of naive T cells into Th17 and Th1 cells (234, 235). The above findings indicate that aspirin may play a role in the treatment of PE by targeting T cells.

### 9.9 Antioxidants

Elevated levels of indicators for oxidative stress and reduced levels of antioxidants (such as vitamin E, vitamin C, and lycopene) in women with PE indicate that oxidative stress markers are significantly involved in the development of PE (236–238). AC-11 (AC-11^®^, hot-water extract of U. tomentosa) has a potential antioxidant effect (239). The administration of AC-11 resulted in a significant decrease in blood pressure and a significant down-regulation of CD8^+^ T cells and CD8/CD4 ratio in PE mice models induced by angiotensin II, compared with healthy pregnant animals (239). A survey of existing research suggests that the products of the kynurenine (Kyn) pathway have the ability to promote T cell differentiation into Treg cells and trigger the apoptosis of Th1 cells (240). Therefore, the Kyn pathway may be a potential therapeutic target for PE due to the antioxidant effect of its metabolites (240). Moreover, the well-established powerful antioxidant pentoxifylline can mitigate oxidative stress and enhance placentation (241). Its function is to maintain the balance between Th1 and Th2 immunity, reduce Th1-type immune responses, and promote a shift towards Th2 immune reactions (242). A different type of antioxidant, known as heme oxygenase-1 (HO-1), has the ability to stimulate the release of Th2 cytokines (243). Moreover, one study has shown that severe PE is linked to elevated T-cell-endothelium adhesion, and effective antioxidants can prevent this impact (244). Clinically achievable concentrations of antioxidants in the circulation can be attained through oral intake of vitamins E and C, or by intravenous administration of N-acetylcysteine, as demonstrated by this study (244). Accordingly, supplementing with antioxidants for PE patients may be a simple and effective treatment.

### 9.10 Transfer of Tregs to enhance anti-inflammatory response

The promotion of alloantigen-specific Treg cells is a potential preventive and therapeutic strategy for PE. This could be achieved by increasing exposure to paternal antigen in high-risk population. In addition, medications that enhance the activity of Treg cells by targeting immune checkpoints, such as CTLA-4Ig (245), may represent viable alternatives for the treatment of PE. To develop therapeutics that target immune checkpoints, it is imperative to investigate costimulatory and inhibitory molecules that are unique to antigen-specific Treg cells. Additional investigation about Treg cells at the single-cell level may yield valuable knowledge regarding the progression of immunotherapeutic approach for PE.

In rodent models of PE, proof-of-concept trials have already demonstrated that PD-L1 Fc (246), CD28 superligand (247), and low dose IL-2 (248, 249) were efficacious biological agents for increasing the percentage and stability of Treg cells. In light of the swift advancements in Treg cell immunology, including the development of numerous intervention therapies and the application of flow cytometry for peripheral blood diagnosis, it is important to investigate Treg cell therapy in high-risk women. Prior to or in the early phases of pregnancy, it is essential to develop diagnostic tools and treatment modailities that can halt the progression of disease and prevent fetal or placental damage. Regarding the “window of opportunity” for timely and effective PE treatment, since Treg cells play an essential role in implantation and placentation during early pregnancy, initiating treatments before conception or as soon as pregnancy is identified is the optimal choice (10).

## 10 Conclusions

There is a limitation in this review due to the insufficient distinction between early-onset and late-onset PE T cell profiles, which arises from the lack of specific studies. In conclusion, a decreased maternal tolerance to paternal antigens is associated with preeclamptic pregnancy. This decrease can be attributed to several factors, including compromised placentation, inadequate oxygen supply to the placenta, heightened activation of the immune system both locally and systematically, a bias to inflammatory T cell response, and increased inflammation. Notwithstanding the copious body of evidence demonstrating the substantial impact of T cell immunity in PE, insufficient endeavors persist to implement these discoveries and improve the standard of care for women afflicted with this obstetric syndrome. As a result, subsequent investigations ought to strive to generate outcomes and frameworks with pragmatic implications, thereby advancing PE prevention, detection, and management.
